# Intrinsic S phase checkpoint enforced by an antiproliferative oncosuppressor cytokine

**DOI:** 10.1038/s41417-021-00397-3

**Published:** 2021-11-04

**Authors:** Livio Mallucci, Valerie Wells

**Affiliations:** 1 Faculty of Life Sciences and Medicine, King’s College London, School of Cancer and Pharmaceutical Sciences, Guy’s Campus, London, SE1 1UL UK; 2 NYU London, 6 Bedford Square, London, WC1B 3RA UK

**Keywords:** Cell biology, Targeted therapies

## Abstract

The cell cycle is strictly programmed with control mechanisms that dictate order in cell cycle progression to ensure faithful DNA replication, whose deviance may lead to cancer. Checkpoint control at the G1/S, S/G2 and G2/M portals have been defined but no statutory time-programmed control for securing orderly transition through S phase has so far been identified. Here we report that in normal cells DNA synthesis is controlled by a checkpoint sited within the early part of S phase, enforced by the βGBP cytokine an antiproliferative molecule otherwise known for its oncosuppressor properties that normal cells constitutively produce for self-regulation. Suppression of active Ras and active MAPK, block of cyclin A gene expression and suppression of CDK2-cyclin A activity are events which while specific to the control of a cell cycle phase in normal cells are part of the apoptotic network in cancer cells.

## Introduction

Checkpoint mechanisms that in normal mammalian cells dictate order and timing during progression from one cell cycle phase to the next to ensure that events critical to genome replication are completed with fidelity have been attracting special interest as perturbations of the related biochemical processes may lead to biological disorders, which include cancer^1–3^. Yet, while much is known on mechanisms of checkpoint control at the G1/S, S/G2 and G2/M portals^1–9^, their relationship with cancer remains fundamentally conceptual. Surprisingly, whether during S phase, a very important stage for the temporal order of DNA replication and genome integrity, DNA synthesis is under programmed checkpoint control remains a fundamental biological question whose impact extends to cancer. A checkpoint monitoring the end of DNA replication has been proposed^6^ but no checkpoint effectors that coordinate timed transition through S phase have been identified. Here we have utilised the 15 kD beta-galactoside-binding protein (βGBP) an antiproliferative cytokine that operates through mechanisms that involve high-affinity receptor binding (Kd ~ 1.5 × 10^−10^ mol/L)^10^ and molecular interactions leading to the functional inhibition of class 1A and class 1B PI3K catalytic subunits^11^. Consequent downregulation of PI3K activity, suppression of Ras-GTP loading and block of ERK activity are conditions that while reversible in normal cells, by blocking the ability of cancer cells to proliferate and by impairing their ability to survive can block oncogenicity.

Based on the evidence that βGBP is an effector molecule that normal cells use for self-regulation^10,12^ and an enforcer of an S phase cytostatic block that drives cancer cells to apoptotic death^13–17^ while instead enforcing reversible arrest in normal cells^10^, we hypothesized that under normal growth conditions βGBP may regulate transition through S phase. Here we report that in secondary cultures of murine embryonic fibroblasts a statutory checkpoint is indeed switched on within a defined time-window set in the early part of S phase via downregulation of signalling that while specific to the control of S phase in normal cells are downstream effectors of apoptotic cell death in cancer cells^18,19^.

## Results

**Fig. 1:**
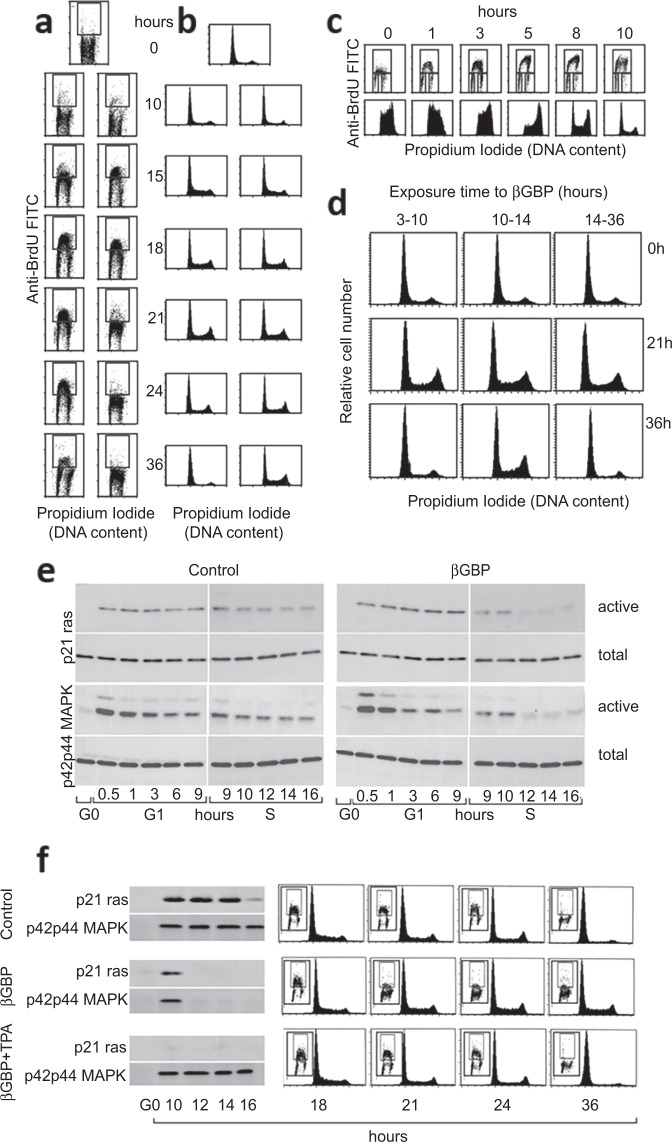
Effect of βGBP on DNA synthesis, cell cycle advance and Ras/MAPK activation. **a** DNA synthesis assessed by BrdU uptake (10 μM) in control cells (left column) and in cells treated with βGBP 2 × 10^−8^ M (standard dose) added at hour 3 after seeding in all experiments (right column). **b** Population distribution of cells in (**a**). **c** Resumption of DNA synthesis (upper panels) and cell cycle advance (lower panels) after negation of βGBP by 10 μl ml^−1^ neutralising monoclonal antibody. **d** Effect of βGBP on cell cycle advance according to exposure time: hour 3 to 10, no effect (left panels); hour 10 to 14, arrest in S phase (central panels); hour 14 and left throughout, no effect (right panels). Murine C57/black embryonic fibroblasts prepared as in ref. ^10^. Images are one representative experiment of several. **e** Western blots of p21Ras and p42/p44 MAPK proteins. **f** Left panels: Western blots; right panels: DNA synthesis and cell cycle advance. Upper panel row: control cells, cell cycle completed. Central panel row: cells where active Ras and active MAPK have been negated, cell cycle block. Lower panel row: cells where only MAPK is operative, cell cycle completed. TPA (1 μM) added at hour 10.

To determine whether under normal growth conditions S phase is under checkpoint control we used secondary murine embryonic fibroblasts, chosen as a paradigm of normal cells cultured to maintain a high degree of population uniformity while undergoing transition through the cell cycle^10^ and have made use of the recombinant form of the human βGBP molecule, which is an autocrine negative growth effector. To gain information on whether βGBP has effect in S phase we first looked at DNA synthesis. Parallel analysis of untreated cells (Fig. 1a left column and 1b left column) and of cells treated with βGBP (Fig. 1a right column and 1b right column) shows that in marked difference with the control population which had transposed into G1 upon completion of a full cycle (hour 36), DNA synthesis and cell cycle progression, that in the treated cells had ab initio progressed in parallel with the controls, had come to a halt at hour 24. To analyse the nature of the impact, βGBP-arrested cells were treated at hour 36 with a βGBP-neutralising monoclonal antibody which abolishes βGBP function^10^. Figure 1c shows that negation of βGBP function led to prompt resumption of DNA synthesis (upper panels) and resumption of S phase progression (lower panels). Repetitions of these experiments confirmed the punctuality with which DNA synthesis and cell cycle advance came to be arrested and to be resumed. To determine whether such an effect was in line with the time-related specificity of a checkpoint we added βGBP and negated its presence at different time intervals in pulse-like experiments. Figure 1d shows that βGBP was effective in enforcing S phase arrest only when present within a 4-h time-window set within the initial part of S phase between hour 10 and hour 14 (central panels), but not otherwise. Outside this defined time-space βGBP did not impede the cells’ ability to complete their full cycle and to proceed into a 2n DNA state (G0/G1). We therefore investigated whether past the hour 10 barrier the active state of Ras and MAPK which are prime effectors of proliferative activity, had been affected. For this, we assessed Ras-GTP loading by the ability of p21^ras^ to bind the Ras binding domain of Raf-1^20^ and measured the phosphorylation state of p42 and p44 MAPK by antibody recognition of the dual phosphorylated protein^21,22^. Figure 1e shows that while in the control population Ras and MAPK were continuously active, there was no significant evidence in the cells treated with βGBP of active Ras and active MAPK beyond the hour 10 barrier. Next, given that block of DNA synthesis coincided with block of Ras/MAPK expression, to ascertain how the arrest of DNA synthesis related to the repression of proliferative signalling, we confronted the active state of Ras and the active state of MAPK to the extent of DNA synthesis under three different conditions: in untreated cells, in cells where Ras and MAPK activity had been negated by βGBP treatment, and in cells where after Ras and MAPK negation, active MAPK, but not active Ras, was re-instated at hour 10 by the addition of TPA (12-tetradecanoyl phorbol-13-,acetate), a phorbol ester which bypasses Ras and activates MAPK via protein kinase C^22^. Figure 1f shows that while, expectedly, in the control cells (upper panels) DNA synthesis and cell advance progressed into a G0/G1 state, in cells where active Ras and active MAPK had been negated (central panels) DNA synthesis and S phase transition had been arrested. On the other hand, in meaningful contrast, in cells where Ras activity was excluded but MAPK activity reinstated by TPA (lower panels), DNA synthesis and S phase transition proceeded unimpeded into G0/G1.

**Fig. 2:**
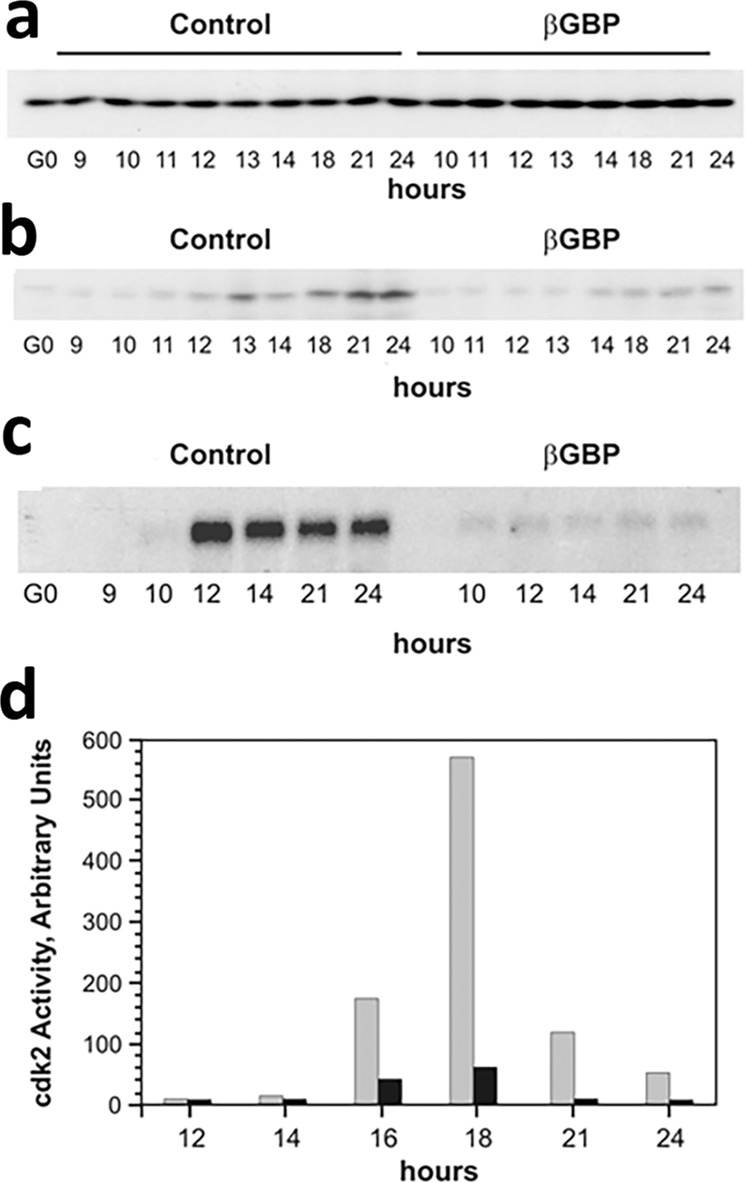
Effect of βGBP on CDK2 protein, cyclin A protein, cyclin A gene and cyclin A-CDK2 activity. **a** Western blot of CDK2; **b** western blot of cyclin A; **c** northern blot of cyclin A mRNA in lysates from 5 × 10^6^ cells. 20 μg RNA run on 1.5% formaldehyde −0.25% agarose gels and hybridised with cyclin A2 cDNA at a stringency of 0.2x SSPE, 0.1% SDS at 50 °C. **d** Cyclin A-CDK2 activity. CDK2 immuno-precipitated and assayed for kinase activity using histone H1^28^. White histograms: control cells; black histograms: cells treated with βGBP. Values are means of triplicate experiments. *P* values < 0.05 versus controls. Loading control reference: CDK2 protein.

Control mechanisms that regulate transit through S phase include those operated by the cyclin A-CDK2 complex whose activity is required for initiation and completion of DNA replication^23,24^. Thus, we investigated whether along with the loss of MAPK activity and the arrest of DNA replication (Figs. 1e and 1a), growth restriction consequent to βGBP treatment included changes in the expression of the cyclin A-CDK2 complex and its activity. We found (Fig. 2a–d) that βGBP treatment did not affect CDK2 protein expression whose levels remained constantly similar regardless of cell cycle stage and regardless of treatment (panel a). By contrast, we found that while cyclin A protein levels had gradually increased throughout S phase in the control cells, there was no evidence in the βGBP-treated cells that significant amounts of the cyclin A protein had been produced (panel b) and no evidence that sufficient CDK2 activity had been made available (~1/10 of control levels at the apex of activity, panel d black histograms) as, as indicated by the revealed absence of mRNA expression, no cyclin a gene activation had occurred (panel c).

## Discussion

Our evidence identifies an intrinsic S phase checkpoint enforced by an antiproliferative cytokine that by controlling basic cellular processes proposes strategies to combat cancer. The data presented argue that the βGBP molecule is an inbuilt regulator of mechanisms that control DNA synthesis prior to the completion of S phase through a process distinct from those activated in response to DNA damage whether externally or internally generated^25–27^, an event that can occur at any time during the cell cycle, βGBP enforcement is programmed to be triggered at a defined time and within a defined window set at the beginning of S phase. Differently from the response to DNA damage which is brought about by specific repair kinases^25–27^, βGBP operates through downregulation of canonical proliferative signals. Suppression of active Ras and active MAPK, block of cyclin A gene expression and suppression of CDK2-cyclin A activity are prime events that lead to the arrest of DNA synthesis. Because of the physiological context of our study, based on normal cells and on an intrinsic growth inhibitor that cells use for self-regulation, we refer to the process here described as an intrinsic S phase checkpoint and put forward that under normal growth conditions a physiologically imposed pause prior to the end of S phase may provide time for DNA screening, error recognition and error mending to secure accurate DNA completion and genome integrity before mitosis. Further to this newly emerged physiological aspect, while it is of interest that by controlling the cell replicative balance βGBP implicitly contributes to opposing the emergence of cancer, it is of particular interest that its mechanistic enforcement of inhibitory signals and consequent cellular blockade, which in normal cells is reversible, in cancer cells are recognised effectors of apoptotic death^18,19^. These traits, that confer oncosuppressor properties to a physiological molecule, suggest strategies that nature uses to combat cancer. They highlight the potential importance of approaching cancer by natural means and provide the rationale for understanding how a process that naturally controls cell proliferation has extended anti-cancer potentials.
